# Sorption and desorption kinetics of PFOS to pristine microplastic

**DOI:** 10.1007/s11356-021-15923-x

**Published:** 2021-08-18

**Authors:** Bettie Cormier, Flora Borchet, Anna Kärrman, Marta Szot, Leo W. Y. Yeung, Steffen H. Keiter

**Affiliations:** 1 Man-Technology-Environment Research Centre, School of Science and Technology, Örebro University, Fakultetsgatan 1, 701 82 Örebro, Sweden; 2 Bordeaux University, EPOC, UMR CNRS 5805, Avenue des Facultés, 33400 Talence, France; 3 RWTH Aachen, Institut für Umweltforschung (Biologie V), Worringerweg 1, 52074 Aachen, Germany

**Keywords:** Microplastics, Polyethylene, Sorption, PFOS

## Abstract

The sorption processes of persistent organic pollutants on microplastics particles are poorly understood. Therefore, the present study investigated the sorption processes of perfluorooctanesulfonate (PFOS) on polyethylene (PE) microplastic particles (MPs) which are representing a prominent environmental pollutant and one of the most abundant microplastic polymers in the aquatic environment, respectively. The focus was set on the investigation of the impact of the particle size on PFOS sorption using four different PE MPs size ranges. The sorption kinetics for 6 months was studied with one selected size range of PE MPs. Besides, the desorption of PFOS from PE MPs under simulated digestive conditions was carried out by using artificial gut fluid mimicking the intestinal juice of fish. The investigation of the size effects of particles over 6 months demonstrated a linear increase of PFOS concentration sorbed onto PE with a decrease of the particle size. Thus, our findings implicate efficient sorption of PFOS onto PE MPs of different sizes. The results showed that PFOS desorbed from the PE MPs into the artificial gut fluid with a rate of 70 to 80%. Besides, a longer exposure of PE MPs to PFOS leads to a higher concentration adsorbed by PE MPs, which may favor the ingestion of higher concentration of PFOS, and thus represents a higher risk to transfer relevant concentrations of PFOS during digestion.

**Supplementary Information:**

The online version contains supplementary material available at 10.1007/s11356-021-15923-x.

## Introduction

The use of plastic has become unavoidable in our society which is directly related with an increasing global plastic production (Geyer et al. 2017). The need for plastics is evident with many societal benefits, which offer the development of new technologies, improved consumer health, and reduced transportation costs (Andrady and Neal 2009; Thompson et al. 2009; Zarfl and Matthies 2010). Besides numerous benefits of plastic products, it becomes obvious that plastics have become one of the major impacts on the environment (Zarfl and Matthies 2010). Plastics may persist and accumulate in the oceans (Law and Thompson 2014) as either macroplastic (> 5 mm), microplastic (MPs; < 5 mm), or nanoplastic (>1μm) (Jambeck et al. 2015; Peng et al. 2020). MPs exist in two types: primary MPs particles consist of plastic granules that are produced on purpose, e.g., in cosmetics but also from the waste of plastic production, whereas secondary MPs are known as small plastic fragments derived from larger macroplastics (Derraik 2002; Thompson et al. 2004). Ubiquitous occurrence of MPs has been observed in oceans, even in the polar areas and in the deep sea (Obbard et al. 2014). Cozar and collaborators (2014) studied 141 water samples and demonstrated the omnipresence of MPs showing that 88% of the water samples contained MPs, with large variation in concentrations. Through different degradation processes, plastic fragments are dispersed in the ocean and converged in gyres (Law and Thompson 2014; Eriksen et al. 2014). Therefore, the abundance of MPs has increased over the last few decades (Barnes et al. 2009). On the other hand, trends in macroplastic accumulation in the marine environment are not uniformly increasing, while the average size of plastic particles seems to be decreasing (Eriksen et al. 2014).

Among all polymers, polyethylene (PE; -(-CH_2_-CH_2_-)_n_-) represents the polymer with the greatest global production for plastic manufacturing (PlasticsEurope 2018). Dris and co-workers showed that PE was one of the most abundant polymers found in aquatic ecosystems (Dris et al. 2015) caused by the extensive use of plastic and improper waste management. In addition, the use of PE as passive sampler demonstrated the ability of PE to concentrate chemicals (Huckins et al. 1990). Besides its extensive use as passive samplers for hydrophobic organic pollutants, PE has been commonly studied as one of the reference polymers to investigate chemical sorption to MPs (Lee et al. 2014; Hüffer and Hofmann 2016; Li et al. 2018; Xu et al. 2018).

Plastic polymers are commonly considered biologically inert and, thus, non-toxic. However, plastic production includes additives such as plasticizers to improve resistance or flexibility, dyes, or fire retardants, and these additives can be toxic (Barnes et al. 2009; Lithner et al. 2011). In addition to the potential toxicity caused by the numerous plastic additives, MPs offer a surface where many waterborne pollutants can be adsorbed, including aqueous metals (Rochman et al. 2014) and persistent organic pollutants (POPs) (Rios et al. 2007; Rochman et al. 2013). In the marine environment, such chemicals are typically found at their highest concentrations in the surface microlayer, where MPs are abundant as well (Ng and Obbard 2006; Rios et al. 2007; Teuten et al. 2007). Thus, MPs are hypothesized to act as vectors and carriers for a wide range of pollutants in the marine environment (Thompson et al. 2004; Barnes et al. 2009; Andrady 2011; Koelmans et al. 2016). The equilibrium kinetics of contaminants on MPs not only depends on the inherent properties of the chemicals, but also on the size, density, and quality of the MPs (Wang et al. 2015, 2019).

In recent years, per- and polyfluoroalkyl substances (PFASs) have attracted attention due to their global occurrence in the environment and their toxicity to aquatic organisms (Huang et al. 2010; Ahrens and Bundschuh 2014; Keiter et al. 2016). Perfluorooctanesulfonate (PFOS), a commonly found PFAS in the environment, has been listed as a POP in Annex B of the Stockholm Convention in 2009 (Paul et al. 2009). PFOS has been restricted due to its bioaccumulation and biomagnification in organisms and food webs, respectively (Martin et al. 2003; Powley et al. 2008; Houde et al. 2008). PFOS has been shown to be ubiquitously present in different ecosystems (Zareitalabad et al. 2013; Ahrens and Bundschuh 2014), in wildlife up to ng/g to μg/g levels (Giesy and Kannan 2001), and human blood at ng/mL levels (Olsen et al. 2005; Kärrman et al. 2006). Concentrations in soil were shown to reach up to 230 μg/g in highly contaminated sites where firefighting foams were used (Baduel et al. 2015). Although PFOS is well-studied in different environmental compartments, a limited number of studies have investigated PFOS in combination with MPs. Wang et al. (2015) studied the sorption of PFOS onto different types of polymers and demonstrated a linear sorption as well as a higher sorption affinity to PE than to polyvinyl chloride (PVC) and polystyrene MPs. However, the impact of the particle size on the sorption behavior of PFOS was not investigated. Bakir et al. (2014) investigated the sorption and desorption of perfluorooctanoic acid (PFOA) on PE and PVC MPs. The results showed a greater desorption using a digestive gut surfactant compared to seawater alone.

The present study was part of the Joint Programming Initiatives Ocean project EPHEMARE (ecotoxicological effects of microplastics in marine ecosystems), which aimed to investigate the distribution, fate, and toxicity of MPs. A major part of the project was to investigate the role of MPs as a vector of pollutants; therefore, PFOS was sorbed on PE MPs of different size ranges. Moreover, to describe the potential desorption of PFOS after sorption processes, a desorption experiment using an artificial gut fluid (AGF) has been performed to evaluate the potential desorption of artificially spiked PFOS from PE MPs. As part of EPHEMARE project, the desorption experiment aimed for a better understanding of what might happen after ingestion of PE MPs spiked with PFOS by fish. For the sorption experiment, we tested two concentrations of PFOS: a low concentration representing environmental relevant level and a high concentration level representing a level which may cause toxic effects. The present study also investigated the sorption processes between PFOS and PE microparticles using different size range that may cover a wide range of PE MPs found in the ocean (Ng and Obbard 2006; Barnes et al. 2009).

## Materials and methods

### Materials and chemicals

Four size ranges of PE MPs were used as the sorbents for the entire EPHEMARE project. Dry powder of PE MPs were purchased from Micro Powders (New York, USA) and provided to all partners of the JPI Oceans project EPHEMARE by Marina Albentosa (Instituto Español de Oceanografía Centro Oceanografico de Murcia, Spain). The provider indicated that particles were composed by non-uniformly shaped particles with a density of 0.96 g/cm^3^ at 25 °C. PE MPs were sieved to size range of 125–500 μm, 20–25 μm, 11–13 μm, and 4–6 μm, and particles were previously characterized using an electronic Coulter Counter (Multisizer III from Beckman Coulter Counter) (Fernández and Albentosa 2019; Gambardella et al. 2019). Sea salt was obtained from Red Sea Fish Pharm LTD., Israel, and sodium taurocholate was from Merck, Germany (NaTC, CAS 345909-26-4). Perfluorooctanesulfonate (PFOS; solid powder; IUPAC: 1,1,2,2,3,3,4,4,5,5,6,6,7,7,8,8,8-heptadecafluorooctane-1-sulfonic acid, CAS 2785-37-3; purity ≥ 98%) and ammonium solution (NH_4_OH) were purchased from Sigma-Aldrich (Stockholm, Sweden) and Fischer Scientific (Ottawa, ON, Canada), respectively. All other chemicals and reagents were purchased at the highest purity available from Sigma-Aldrich (Stockholm, Sweden), unless stated otherwise. Analytical standards including PFOS technical mixture and mass labelled standards of PFOS (^13^C_8_ PFOS and ^13^C_4_ PFOS) were purchased from Wellington Laboratories (Guelph, ON, Canada).

### Sorption of pollutants onto PE MPs particles

Three sorption experiments were performed investigating the role of PE MPs as vector for PFOS. In the first experiment, the sorption process of two PFOS concentrations over 7 days onto PE MPs was investigated and measured at three time points (2 days, 5 days, and 7 days). Besides, this process allowed the production of high amounts of spiked PE MPs for all EPHEMARE partners. The three time points were selected based on previous other studies (Wang et al. 2015). For all size ranges used, PFOS concentration in water was experimentally adapted to the different size ranges to obtain similar final concentration ranges of PFOS on the different PE MPs (Table 1). The spiked PE MPs were distributed among all partners of the EPHEMARE project.

**Table 1:** PFOS concentrations in water for the high and low concentrations of sorption experiments

	High [PFOS] in water	Low [PFOS] in water
PE (500–125 μm)	600 mg/L	0.6 mg/L
PE (25–20 μm)	30 mg/L	0.03 mg/L
PE (13–11 μm)	20 mg/L	0.02 mg/L
PE (6–4 μm)	10 mg/L	0.01 mg/L

The sorption of PFOS on different size ranges of PE MPs (125–500, 20–25, 11–13, and 4–6 μm) was performed using 1-L polypropylene (PP) bottles (Lamaplast; Sesto San Giovanni, Italy). Twenty-five milligrams of PE MPs, 500 mL of MilliQ water (Millipore, Billerica, MA, USA), and adjusted concentrations of PFOS (Table 1) were mixed, e.g., mg/L and μg/L for high and low concentrations, respectively, in the PP bottles, and placed on a rotary shaker (Heidolph Instruments GmbH & CO. KG, Schwabach, Germany) for up to 7 days at 20 rpm. After 7 days, PE MPs particles were filtered using 1 μm Whatman® glass microfiber filter (GE Healthcare Life Sciences; Uppsala, SE). Subsequently, the particles were rinsed three times with 5 mL of MilliQ water. The sorption experiment was performed in three replicates. Blank experiments were performed as control to evaluate losses of PFOS through all processes, e.g., sorption to PP bottles, filtration, and rinsing. PFOS contamination was also assessed and accounted for all experiments. In parallel, we measured whether the PE MPs were contaminated with PFOS by the different solvents, items, and the laboratory equipment that were used for the sorption experiments. Investigations were conducted in two different laboratory rooms at MTM Research Centre referred as shaking room and extraction room. For the identification of potential sources of PFOS contamination, different items were investigated, e.g., methanol (HPLC and LC/MS/MS grades, Sigma-Aldrich, Sweden), pipets, ultrasonication bath, centrifuge, and standards. For each measurement, two independent replicates were prepared. Each item tested was new, and the solvent bottles were freshly opened. All chemical analyses were performed as described below.

### Sorption kinetics

The aim of the second experiment was to study the long-term sorption kinetics of PFOS onto PE MPs. This experiment was conducted in triplicate in 50-mL PP centrifuge tubes filled with 50 g/L of PE MPs (125–500 μm PE), 50 mL of MilliQ water, and 200 mg/L of PFOS. Tubes were continuously agitated on a rotatory shaker at 20 rpm for 180 days. Samples were filtered as previously described and collected after 2, 7, 30, 90, and 180 days, and PE MPs were extracted and analyzed as described in the chemical analysis section. Water samples were analyzed after spiking with ^13^C_4_ PFOS standard before instrumental analysis.

### Size effect of PE MPs on PFOS

The third sorption experiment assessed the effect of the particles size range on the PFOS sorption, by using 4 different particle sizes with the same protocol and the same initial amount of PFOS. The sorption isotherm experiment was performed for 7 days. Containers for the isotherm experiments were alike the ones used for the sorption kinetics. Tubes contained 0.1 g of PE MPs (4–6, 11–13, 20–25, or 125–500 μm) and 40 mL of MilliQ water solution with different PFOS concentrations ranging from 0, 25, 50, 75, and 100 μg/L, PE MPs. Tubes were shaken at 20 rpm and kept at 20 ± 1 °C for 7 days. All experiments were conducted as three replicates.

### Desorption of PFOS from PE MPs by artificial gut fluid (AGF)

A desorption experiment was performed to investigate the desorption process of PFOS from PE MPs using a gut surfactant. The most commonly used protocol was developed to mimic the physiological conditions in the marine lugworm *Arenicola marina* (Voparil and Mayer 2004) containing a model protein (bovine serum albumin, BSA) and sodium taurocholate as the only bile salt, which is a taurine-conjugated C24 bile salt and actually produced in vertebrates (Haslewood and Tökés 1969). In the present approach, no proteins were added to the AGF to focus on the role of bile during digestion and to eliminate an additional factor of uncertainty. Among teleost fish, approximately 60% of species produce no other than C24 bile salts for their digestive juices (Hofmann et al. 2010). This promotes sodium taurocholate as a suitable model bile salt in AGF of Atlantic Cod (*Gadus morhua*), providing a low-cost and feasible approach to assess the desorption behavior of MPs-associated toxicants in a broad range of species. Based on the protocol of a previous study (Voparil and Mayer 2004), AGF was prepared by resolving 35‰ sea salt and 15 mM sodium taurocholate in MilliQ water, and pH was adjusted to 6.7 by using 1 M hydrochloric acid (Sigma-Aldrich, Stockholm, Sweden) (Grosell et al. 2001). The digestion has been performed according to the model used by Koelmans et al. (2014). The scenario was performed to simulate the digestion of 3300 g Atlantic Cod (*Gadus morhua*) which ingests 0.0126 g food/g bodyweight per day (1.26%). Since our study focused on the uptake of PE MPs by food, quantities of 0.3% (Jovanović et al. 2018) and 1% (Cormier et al. 2021) of PE MPs related to ingested food per day were chosen. Gut retention time was assumed to be 4 days on average (Daan 1973). Thus, PE MPs (11–13 μm) were spiked with PFOS as previously described for the sorption section (Sorption of pollutants onto PE MPs particles) and were artificially digested in 10 mL AGF in PP tubes in triplicates. Quantities of 503 mg of PE MPs and 1663 mg of PE MPs were digested to represent a digestion of 4 days, for 0.3% and 1% of PE MPs, respectively. Controls (*n=2*) consisted of 10 mL pure AGF and 10 mL AGF containing 1% PE MPs. Samples were incubated in darkness for 4 days on a rotary shaker at 20 rpm at 20 ± 1 °C. Samples were filtered by using a glass fiber filter on a funnel connected to vacuum as described before. Exposure tubes and particles were rinsed with 5 mL and 1 mL of MilliQ water, respectively.

### Chemical analysis of PFOS

#### Sorption experiments

##### Analysis of PFOS in water

An aliquot of the water samples were spiked with 1 ng mass-labelled ^13^C_4_ PFOS (Wellington Laboratories Inc., Canada) and filtered using a PE syringe (Norm-Ject®; Henke Sass Wolf, Tuttlingen, Germany) with a filter of 0.2 μm (13 mm, 0.2 μm AcrodiscGHP; Pall, Dreieich, Germany). The water extracts were transferred to LC vials for analysis. In the LC vials, it contained 300 μL of the water aliquot, and 200 μL of organic mobile phase (2 mM ammonium acetate in methanol) was added. The LC vial was vortexed for 30 s and ready for instrumental analysis.

##### Analysis of PFOS sorbed on PE MPs

Chemical analysis of PFOS on PE MPs has been conducted according to Eriksson et al. (2016) with some modifications. In a 15 mL PP tube, 0.3 g of PE MPs was spiked with 1 ng mass-labelled ^13^C_4_ PFOS (Wellington Laboratories Inc., Canada) and extracted in 2 mL of methanol (MeOH HPLC grade, > 99.9% purity, Fisher Scientific, New Jersey) by ultrasonication for 15 min followed by 10 min of centrifugation at 7000 rpm. The extraction procedure was repeated twice, and extracts were combined. Extracts were then filtered using a PE syringe and a filter of 0.2 μm (13 mm, 0.2 μm AcrodiscGHP; Pall, Dreieich, Germany), and then, the extract was concentrated to 200 μL under a gentle stream of nitrogen. The 200 μL extract was then transferred to the LC vial and spiked with 2 ng of mass-labelled ^13^C_8_ PFOS (stock solution of 0.2 ng/μL). Finally, 300 μL of aqueous mobile phase (2 mM ammonium acetate) was added. The LC vial was vortexed for 30 s and ready for instrumental analysis.

#### Desorption experiment, PFOS in AGF

Chemical analysis of AGF used solid phase extraction (SPE). First, 10 mL of AGF in triplicates were adjusted to pH 4 using acetic acid (97%, Sigma-Aldrich, Sweden) and spiked with 1 ng mass-labelled ^13^C_4_ PFOS (Wellington Laboratories Inc., Canada). SPE cartridges (Oasis® WAX 150 mg 6 cc, Waters®) were preconditioned with 4 mL of 0.1% of NH_4_OH (25%, Fisher Scientific) in methanol, followed by 4 mL of methanol and finally 4 mL of MilliQ water. Then, samples were loaded on the cartridges. The cartridges were washed using 20 mL of MilliQ water, 4 mL of ammonium acetate buffer (pH 4), and 4 mL of methanol. Target compound was eluted using 4 mL of 0.1% NH_4_OH in methanol, and the extracts were evaporated down to 200 μL by nitrogen stream. One nanogram of mass-labelled ^13^C_8_ PFOS was added (stock solution of 0.2 ng/μL), and samples were transferred to LC vial containing 300 μL of 2 mM ammonium acetate prepared in MilliQ water. Analysis of PE MPs followed the method as described above (“Analysis of PFOS sorbed on PE MPs”).

### Instrumental analysis

Analyses were performed on an Acquity UPLC system coupled to a Xevo TQ-S quadrupole MS (Waters, Milford, USA) operated in negative electron-spray ionization mode (ESI-). Target compounds comprised linear PFOS (L-PFOS) and six mono-methylated, branched PFOS isomers (1*m*-PFOS, 6/2*m*-PFOS, 3/4/5*m*-PFOS). The term “PFOS” refers to the sum of all quantified isomers. Separation of PFOS isomers was achieved using an Acquity BEH C18 column (2.1 × 100 mm, 1.7 μm). A gradient program delivered two mobile phases of (A) 2 mM ammonium acetate in water/methanol 70:30 and (B) 2 mM ammonium acetate in methanol. Column temperature was kept at 50°C with a flow rate set to 0.3 mL/min. The LC operated with an injection volume of 10 μL and the elution gradient started with 100% A and was increased up to 100% B in 14 min and then decreased to 0% with solvent B at 14.2 min, followed by equilibration until 17 min. MRM transitions for PFOS isomers are provided in the SM (Table 1, SM). A guard column (isolator column, Waters Corporation, Milford, USA) was inserted between the pump and the injector to prevent contamination from the system. MassLynx™ V 4.2 Software (Waters®) was used for data evaluation. Quantification of PFOS isomers was done using the internal standard method with ^13^C_4_ mass labelled PFOS applying a 5-point calibration curve (Wellington Laboratories Inc., Canada).

### Quality assurance and quality control (QA/QC)

The quality assurance and quality control measures taken in this investigation include (i) measures to avoid or lower contamination of PFOS during the course of the experiment; (ii) procedure blanks consisting of MilliQ water without any sample matrix following the same extraction procedure as samples to assess if there was any contamination during the extraction procedures; (iii) sample recovery based on the amounts of the mass-labelled ^13^C_4_ PFOS spiked before extraction (1 ng) and mass-labelled ^13^C_8_ PFOS spiked after extraction (2 ng) to assess extraction efficiency of the method; (iv) sample analysis were conducted in triplicates to assess precision; and (v) evaluation of method detection limit (MDL) as the lowest concentration that was observed in the calibration curve when no contamination was detected; otherwise, the MDL was calculated as mean blank concentration with the addition of three times the standard deviation. To avoid contamination from the LC system that might interfere with the samples, an isolator column (isolator column, Waters) was inserted between the pump and the injector. All the glassware and consumables had been rinsed with MeOH. Background contamination in PE MPs was also evaluated using 1 g of each PE MPs size range in triplicates and conducted according to the description above. Background contamination of PFOS in PE was observed in the range of 10–15 pg/g (Table 2, SM), and the spiking was at μg/g levels (Table 1). Background check on the AGF was conducted prior to experiments and to determine any background contamination of PFOS in the pure fluid and to validate the extraction method; no detectable PFOS was found in the AGF. Procedure blanks were included in all batches; no detectable levels of PFOS were found in the procedure blanks. The extraction efficiency of the PE MPs in the sorption experiment was found to be 83–96%. Since no detectable blank was observed, the MDL corresponded to the lowest concentration observed on the calibration curve (10 pg/g).

**Table 2:** PFOS adsorbed on PE particles with high and low concentration of chemicals after 7 days. Concentration mean of 3 independent samples ± SD, *n*=3

	PE particles			
	6–4 μm	13–11 μm	25–20 μm	500–125 μm
High [PFOS]_PE MPs_ (μg/g)	73.6 (± 7.1)	46.3 (± 4.3)	54.0 (± 1.1)	41.2 (± 9.2)
Low [PFOS]_PE MPs_ (ng/g)	22.6 (± 3.6)	30.2 (± 0.5)	20.6 (± 1.6)	63.3 (± 9.1)

### Statistics

The amount of PFOS sorbed per unit mass of PE MPs was analyzed, and a distribution coefficient (Kd, L/kg) for each individual data point within the linear range of sorption isotherms was calculated as *K*_*d*_ = *C*_*m*_/*C*_*e*_, with the concentration sorbed to the PE MPs (C_m_) and C_e_ as the final equilibrium concentration in the water solution. A one-way ANOVA or a non-parametric test, Kruskal-Wallis, was performed on data to statistically analyze the difference of the datasets. Parameters for sorption experiments were determined using Microsoft Excel 2010 and GraphPad Prism 8.1. All analyses were performed as three replicates (experiments), and two injections were analyzed. Uncertainties in results represent deviation of experimental and injection replicates.

## Results and discussion

PFOS and other per- and polyfluoroalkyl substances (PFAS) possess a great chemical stability and specific surface properties (Paul et al. 2009); thus, these compounds were used in numerous products, e.g., varnishes, waxes, firefighting foams, metal plating and cleaning, coating formulations, lubricants, and as a repellent for leather, paper, and textiles (Ahrens and Bundschuh 2014; Paul et al. 2009). PFOS appeared to be one of the main PFAS that has been detected as a contaminant in different matrices, e.g., indoor air, indoor dust, and drinking water (Jian et al. 2017; Stubleski et al. 2017). In the present study, we detected a background contamination of PFOS on PE MPs that originated from methanol and the different equipment used in both laboratory rooms. Methanol used for the sample preparation contained traces of PFOS (0.12 ng/L). The major PFOS contamination occurred most likely during the transfer of MeOH using glass and plastic pipettes, while sonication and centrifugation processes did not cause any PFOS contamination. To prevent contamination from standards, new batches were prepared and revealed an absence of PFOS. In total, the background contamination accounted for 10–15 pg/g on PE MPs. Background contamination may represent a common issue in laboratories. Illing et al. (2014) demonstrated multiple sources of contamination and suggest using passive samplers to characterize temporal and spatial contamination of laboratories. The following presented results are considering the background contamination of PFOS.

### Sorption of PFOS

PFOS concentrations were in the same range for all particle sizes, 41.2–73.6 μg/g and 20.6–63.3 ng/g for high and low concentrations, respectively (Table 2). The initial PFOS concentrations in water were held approximately constant with respect to the particles size, with a concentration to particle size ratio of 0.6 to 0.8. The concentration of PFOS obtained on PE MPs particles at low sorption, e.g., ng/g range, corresponded to environmental concentration that were found in sewage sludge (Loganathan et al. 2007; Becker et al. 2008; Guo et al. 2009), whereas the PFOS concentrations at high sorption concentrations tends to represent a concentration inducing acute toxicity on zebrafish embryos exposed to the pure compound (Ye et al. 2009).

The effect of the particle size on the sorption capacity of PE MPs is poorly understood, but it has been shown that a decrease in particles size, at micron and submicron size, increased the sorption rates of contaminants (Wang et al. 2019). In fact, the sorption to smaller particles is less limited by intraparticle diffusion but also by a larger surface-to-volume ratio and a faster film diffusion due to a thinner boundary layer (Fries and Zarfl 2012; Seidensticker et al. 2019).

In general, the (de)sorption behavior varies depending on sorbent and sorbate (Bakir et al. 2012). For PFASs, it is assumed that hydrophobic and/or electrostatic interactions are two main sorption processes onto organic material (Higgins and Luthy 2006). Differences in sorption efficiencies between PFASs can be attributed to differences in functional groups. For example, the sulfonate group of PFOS bears a stronger polarity than the carboxylic group of PFOA (Bakir et al. 2014). Also, the smaller size of the carboxylic head of PFOA than the sulfonate head of PFOS led to a greater sorption of PFOS to sorbate (Oliver et al. 2020). The presence of hydrophobic and hydrophilic properties of PFOS allowed sorption to hydrophobic materials, such as soils (Oliver et al. 2020). In the present study, PFOS was successfully sorbed onto PE MPs, and hydrophobic interactions appeared to be the main parameter influencing the sorption processes. Further, PFASs are often amphiphilic substances and, thus, might show a highly substance-specific sorption behavior. To date, studies investigating specifically the sorption behavior of PFASs along with PE MPs are rather scarce.

### Sorption kinetics

Sorption kinetics are a relevant insight into the mechanisms involved in the vectorization of chemicals toward sorbents (Azizian 2004). In general, sorption kinetics using MPs are performed on a small scale, e.g., for hours or days (Zhan et al. 2016; Zhang et al. 2018). In the present study, a long-term sorption experiment was conducted using PFOS to investigate sorption onto PE particles (125–500 μm) over 180 days.

**Fig. 1:**
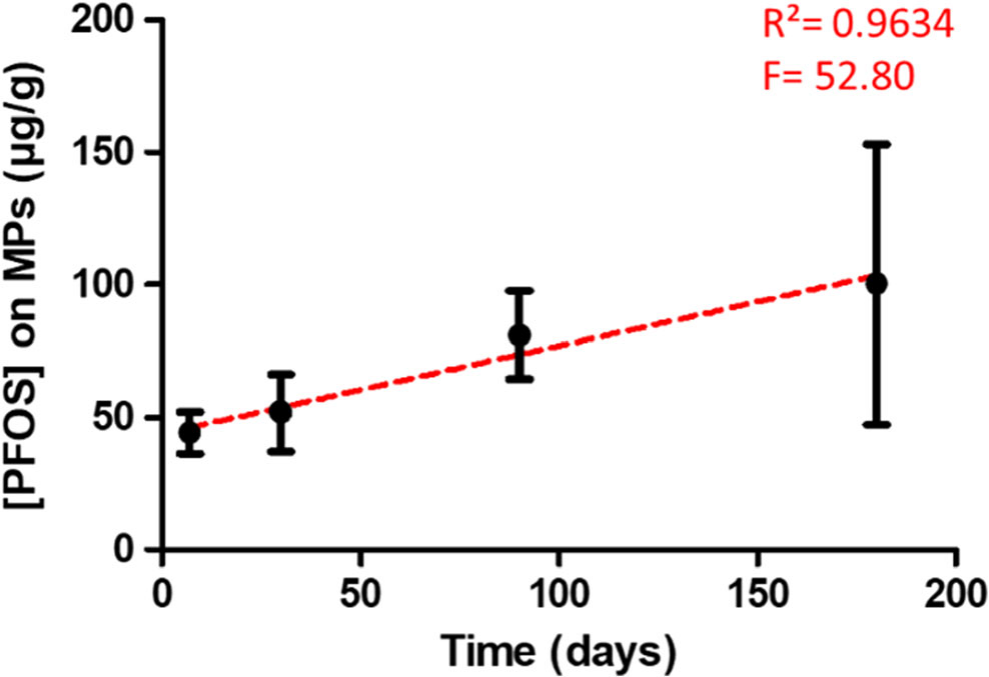
Concentration of PFOS in μg/g, adsorbed on PE MPs (PE 250–500 μm) after 7, 30, 90, and 180 days of exposure to 200 mg/L of PFOS. Mean ± SD; (*n*=3, experiments)

Experimental data were plotted, and a linear regression was performed (*F*=52.80, *R*^2^=0.9635) (Figure 1). The sorption increased over time, and at 180 days, a high standard deviation was observed.

The linearity of the observed sorption is in line with the results of a previous study on PFOS sorption on PE, PS, and PVC MPs (Wang et al. 2015). However, this is the first study that investigated the sorption kinetics of PFOS on PE MPs. Previous studies with activated sludge and carbon nanotubes showed that the equilibrium with PFOS was reached within 11 h and 2 h, respectively (Zhou et al. 2010; Deng et al. 2013). PFOS concentration adsorbed on PE MPs represented 1% up to 2.5% of the initial amount of PFOS introduced into water, after 7 and 180 days, respectively. The limited sorption of PFOS onto PE MPs might be due to various parameters including the use of high initial concentration of PFOS leading to the sorption of PFOS onto plastic materials (lids and tubes) despite previous saturation of the tubes. In addition, the high variation at 180 days might be due to the aging of particles remaining in solution for 6 months and leading to a degradation/modification of PE MPs surfaces with modification of the sorption sites. Indeed, the potential degradation of PE MPs might lead to smaller particles with a bigger surface area leading to an increase of the number of available binding sites for PFOS. The partitioning via hydrophobic interaction and electrostatic interaction seemed to be two potential sorption mechanisms for PFOS on sediments (Higgins and Luthy 2006). In the present study, the anionic form of PFOS was the main ion present in solution; thus, the low sorption rate of PFOS could be explained by electrostatic repulsion from the surface of PE MPs (Wang et al. 2015). In addition, the duality between hydrophobic and hydrophilic properties of PFOS (Krafft and Riess 2015) might lead to hydrophobic interaction between hydrophobic surface of PE MPs and perfluorocarbon chains (Wang et al. 2015).

### Size effect of PE MPs on PFOS

**Fig. 2:**
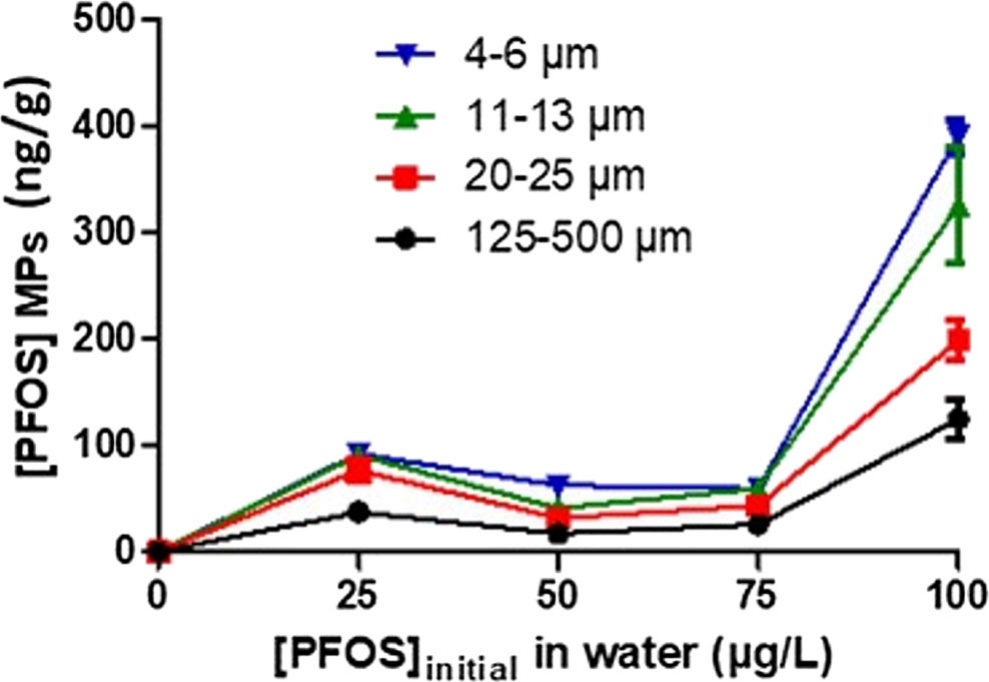
Concentrations of PFOS in μg/g adsorbed on different size particles of PE (4–6; 11–13; 20–25; and 250–500 μm) after exposure to 25, 50, 75, and 100 μg/L of PFOS for 7 days. Mean ± SD; *n*=3

The sorption of PFOS on different sizes of PE MPs is presented, and the equilibrium dissociation constants calculated are shown in Table 3. A tendency can be observed that the sorption efficiencies were shown to be higher for small particles than for bigger particles in all tested concentrations (Figure 2). However, only the highest concentration, 100 μg/L of PFOS, led to significant differences between particle sizes with an increase of sorption efficiency link to the decrease of particles size. Equilibrium dissociation constants (Kd) revealed a greater sorption, while the particle size decreases (Table 3). At 100 μg/L of PFOS, Kd values for PE particles ranged from 20.9 to 62.6 L/kg. A previous study observed similar distribution coefficient of K_d_ = 32.8 L/kg determined for PE MPs (150 μm) and PFOS (Wang et al. 2015). In addition, the decrease of the sorption efficiency of PFOS with an increase of initial PFOS concentration from 25 to 50–75 μg/L (Figure 2) might be explained by saturation of the sorption sites of PE MPs (Wang and Wang 2018). The type of polymer might also affect the sorption rate of a compound. PE particles have a high segmental mobility and free volume which can support the sorption of chemicals (Pascall et al. 2005; Karapanagioti and Klontza 2008). Therefore, differences in particle sizes, inducing a modification of surface areas, could have contributed to the difference in chemical uptake. Numerous studies have shown effects of the particle size of sorbents on the sorption efficiency of different chemicals (Waychunas et al. 2005). Mckay et al. (1982) found that particle size of chitin had no effect or only a little impact on the sorption efficiency of a dye. Chiou and Li (2002) reported an absence of clear effect size with the sorption of dyes onto chitosan beads. No scientific consensus has been highlighted on the effect of the particle size of a sorbent on the sorption efficiency of contaminants. Since this effect of size depending sorption processes on PE MPs barely has been studied yet, it remains unclear if a decrease of particle sizes is inducing an increase of the sorption rate of a contaminant. In addition, external factors such as temperature, pH, salinity, and composition of the water phase (e.g., dissolved organic matter, ion concentrations) can also influence the sorption efficiency of compounds onto MPs (Endo and Koelmans 2019; Wang et al. 2015).

**Table 3:** Distribution coefficient (Kd, L/kg) values of PFOS concentrations for tested PE MPs exposed to 100 μg/L of PFOS for 7 days. Mean ± SD, different letters indicate significant differences (ANOVA, p < 0.05)

	Kd, PFOS (L/kg)
PE 4–6 μm	62.6 ± 3.8^a^
PE 11–13 μm	54.2 ± 6.3^a^
PE 20–25 μm	33.8 ± 5.1^b^
PE 125–500 μm	20.9 ± 4.7^c^

### Desorption of PFOS from PE by AGF

PFOS-spiked PE particles were exposed to AGF to simulate the digestion of contaminated MPs in Atlantic Cod (*Gadus morhua*). Background levels of PFOS in AGF were negligible (2.5.10^−5^ ± 1.1.10^−6^ μg_PFOS_/mL_AGF_). Regardless the quantity of exposed PFOS-spiked particles, desorption was more than 75%. Initial PFOS concentrations (*d* = 0 days) encompassed 0.72 and 2.40 μg_*PFOS*_/ml_*AGF*_ (PFOS sorbed on the particles, Table 4) for 0.3 and 1% spiked particles, respectively. Therefore, 0.55 ± 0.08 μg_*PFOS*_/ml_*AGF*_ and 1.86 ± 0.18 μg_*PFOS*_/ml_*AGF*_ desorbed into the gut fluid of after *d* = 4 days and corresponds to a desorption of 76.8 and 77.6% for 0.3 and 1% spiked MPs, respectively (Table 4).

**Table 4:** Sorbed *versus* desorbed PFOS concentrations before (*d*=0 days) and after (*d*=4 days) exposure to artificial gut fluid (AGF). Two quantities (0.3 and 1%) of PFOS-spiked PE MPs were applied (sorbed PFOS *n*=3, desorbed PFOS *n*=6)

Exposed PFOS-spiked MPs quantity	Sorbed PFOS onto MPs, *d*=0 (μg_PFOS_/ml_AGF_)	Desorbed PFOS into AGF, *d*=4 (μg_PFOS_/ml_AGF_)	% desorption
PFOS PE MPs 0.3%	0.72 ± 0.08	0.55 ± 0.08	76.8
PFOS PE MPs 1%	2.40 ± 0.28	1.86 ± 0.18	77.6

The high desorption of more than 75% from both PFOS-spiked MPs quantities is most likely attributed to molecular properties and chemical interactions between sodium taurocholate and PFOS. Both compounds are amphiphilic consisting of a hydrophobic and a polar functional group (*Figure* *1**, SM*). Thus, the bioavailability of PFOS might be enhanced by the inclusion into bile salt micelles or into their micellular layer (Mazer et al. 1980). For example, a mixture of 30–40% of polychlorinated biphenyls (PCBs) and 57% of lindane was desorbed from the soil after in vitro digestion with chicken bile (Oomen et al. 2000). Another study investigated the digestion of soil contaminated with polycyclic aromatic hydrocarbons (PAHs) and PCBs and revealed a mobilization of PAHs/PCBs after gastro-intestinal digestion up to two times higher than gastric digestion without bile (Hack and Selenka 1996). In the same study, a maximum desorption of 40% was measured within 6 h for PCBs and PAHs. In the present study, PFOS showed even higher desorption of 80% over a period of 4 days. PFOS is more amphiphilic than hydrophobic POPs like PCBs and PAHs. Hence, our results suggest that the desorption efficiency of bile depends on the hydrophobic and/or amphiphilic properties of the particle-bound toxicant. Despite particular affinities between different HOCs and MPs, Bakir et al. (2014) demonstrated a significant increase of desorption in the presence of gut surfactant between 2 and 20 times higher compared to seawater. Similar results were presented by Teuten et al. (2007) for phenanthrene in combination with PE and PP. It is proposed that even though contaminants desorb more slowly from plastics than from sediment, the relative amount desorbing from plastics under physiological conditions might exceed the quantities desorbing from natural sediment particles (Bakir et al. 2014). Considering the comparable long gut retention time in Atlantic Cod as well as chronic exposure to MPs contaminated prey, a desorption of more than 75% suggests a higher threat of MPs to contribute to the PFOS burden of marine fish.

## Conclusion

Sorption behaviors of PFOS, an environmental contaminant on different sizes of PE MPs particles, were investigated. A long-term sorption demonstrated a linear sorption of PFOS over 6 months. A decrease of the particle sizes increased the sorption efficiency of PFOS, due to a higher surface area of smaller particles. Further studies should include different types of polymers (e.g., PS, PP) and more relevant concentrations of MPs and pollutants. Investigations on emerging pollutants together with PFASs in combination with other plastic types are needed since adsorption and desorption behavior depends both on the plastic material and types of pollutants. To the best of our knowledge, the present study is the first to investigate the desorption behavior of PFOS spiked on PE MPs under simulated physiological conditions. We showed that the bile salt is desorbing PFOS from PE MPs within 4 days during artificial digestion. Hence, sodium taurocholate enhanced the bioavailability of PFOS associated to PE MPs. To further investigate the desorption efficiency of sodium taurocholate, desorbed quantities of PFOS should be measured at several time points to determine desorption kinetics. The developed method might be used as an approach to a more appropriate understanding of toxicants desorption from PE MPs during digestion. Future projects should also make use of higher tier studies to evaluate the burden of MPs as vectors for toxic substances as well as the reliability of artificial digestion experiments.

## Supplementary Information

ESM 1 (DOCX 52 kb)

## Data Availability

The datasets used and/or analyzed during the current study are available from the corresponding author on reasonable request.
