# Supplementary material for: Sorption and desorption kinetics of PFOS to pristine microplastic
Source: Environ Sci Pollut Res Int. 2021 Aug 18;29(3):4497–507. doi: 10.1007/s11356-021-15923-x (PMC8741692; doi:10.1007/s11356-021-15923-x)
Supplement: Supplementary file 1 — (DOCX 52 kb) [file 11356_2021_15923_MOESM1_ESM.docx]

**Supplementary material**

Table 1, SM: Details of MRM transitions, cone voltage, and collision energy used for quantification and qualification of PFOS isomers

| PFOS isomers /  co-eluted isomers | parent | Daughters | Cone (V) | Collision (V) |
| --- | --- | --- | --- | --- |
|  | **(m/z)** | **(m/z)** | **(V)** | **(eV)** |
| L-PFOS | 498.97 | 98.96^⁎^  169.03 | 20 | 38  34 |
| 1*m*-PFOS | 498.97 | 98.96^⁎^  169.03 | 20 | 38  34 |
| 6/2*m*-PFOS | 498.97 | 98.96  169.03^⁎^ | 20 | 38  34 |
| 3/4/5*m*-PFOS | 498.97 | 98.96  79.96^⁎^ | 20 | 38  44 |

⁎: quantified product ion

Table 2, SM: Determination of PFOS concentrations in freshly opened solvent ; pipettes and in all steps of the preparation and extraction protocol ; e.g. transfer of MeOH with 3 different plastic or glass pipettes, transfer of MeOH with same glass pipette, sonication bath and centrifuge. (n=2, concentration in pg/mL, mean ± SD)

|  | | PFOS concentration (pg/mL) |
| --- | --- | --- |
| Solvent | MeOH (HPLC grade) | 0.12 (± 0.2) |
|  | MeOH (LC MS grade) | 3.24 (± 0.85) |
| Pipettes | Plastic pipette tips | 0.69 (± 0.15) |
|  | Glass pipette | 0.45 (± 0.15) |
| Entire preparation | *Step*: Transfer plastic pipettes | 5.26 (± 1.28) |
|  | *Step*: Transfer glass pipette | 2.75 (± 1.46) |
|  | *Step*: Transfer of MeOH with same glass pipette | 10.12 (± 4.01) |
|  | *Step*: Sonication | 0.16 (± 0.01) |
|  | *Step*: Centrifugation | 0.21 (± 0.06) |


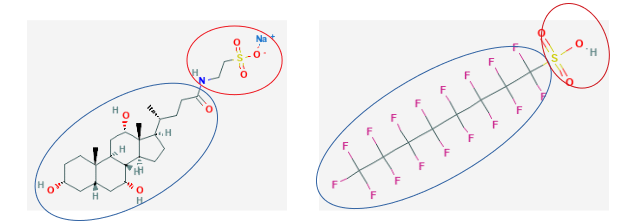
**Figure 1, SM.** Molecular structure of sodium taurocholate (left) and PFOS (right). Hydrophobic chains and polar functional groups are marked in both compounds in blue and red, respectively.
